# Replicative phenotyping adds value to genotypic resistance testing in heavily pre-treated HIV-infected individuals - the Swiss HIV Cohort Study

**DOI:** 10.1186/1479-5876-9-14

**Published:** 2011-01-21

**Authors:** Jan Fehr, Tracy R Glass, Séverine Louvel, François Hamy, Hans H Hirsch, Viktor von Wyl, Jürg Böni, Sabine Yerly, Philippe Bürgisser, Matthias Cavassini, Christoph A Fux, Bernard Hirschel, Pietro Vernazza, Gladys Martinetti, Enos Bernasconi, Huldrych F Günthard, Manuel Battegay, Heiner C Bucher, Thomas Klimkait

**Affiliations:** 1 Division of Infectious Diseases & Hospital Epidemiology, University Hospital of Basel, Petersgraben 4, CH-4031 Basel, Switzerland; 2 Basel Institute for Clinical Epidemiology and Biostatistics, University Hospital of Basel, Hebelstrasse 10, CH-4031 Basel, Switzerland; 3 InPheno AG, Vesalgasse 1, CH-4051 Basel, Switzerland; 4 Department of Biomedicine, Institute for Medical Microbiology, University of Basel, Petersplatz 10, CH-4003 Basel, Switzerland; 5 Division of Infectious Diseases & Hospital Epidemiology, University Hospital, University of Zürich, Raemistrasse 100, CH-8091 Zurich, Switzerland; 6 Swiss National Centre for Retroviruses, Zurich, Winterthurerstrasse 190, CH-8057 Zurich, Switzerland; 7 Laboratory of Virology, University Hospital of Geneva and University of Geneva Medical School, Rue Gabrielle-Perret-Gentil 4, CH-1211 Geneva, Switzerland; 8 Division of Immunology, University Hospital Lausanne, University of Lausanne, Rue du Bugnon 46, CH-1011 Lausanne, Switzerland; 9 Infectious Diseases Service, Department of Internal Medicine, University Hospital of Lausanne, University of Lausanne, CH-1011 Lausanne, Switzerland; 10 Clinics for Infectious Diseases Bern, University Hospital and University of Bern, Freiburgstrasse 4, CH-3010 Bern, Switzerland; 11 Division of Infectious Diseases, University Hospital of Geneva and University of Geneva Medical School, Geneva, Rue Gabrielle-Perret-Gentil 4, CH-1211 Geneva, Switzerland; 12 Division of Infectious Diseases, Cantonal Hospital St. Gallen, Rorschacher Strasse 95, CH-9007 St. Gallen, Switzerland; 13 Institute for Medical Microbiology, Ospedale Civico Lugano, Via Tesserete 46, CH-6903 Lugano, Switzerland; 14 Division of Infectious Diseases, Ospedale Civico Lugano, Via Tesserete 46, CH-6903 Lugano, Switzerland

## Abstract

**Background:**

Replicative phenotypic HIV resistance testing (rPRT) uses recombinant infectious virus to measure viral replication in the presence of antiretroviral drugs. Due to its high sensitivity of detection of viral minorities and its dissecting power for complex viral resistance patterns and mixed virus populations rPRT might help to improve HIV resistance diagnostics, particularly for patients with multiple drug failures. The aim was to investigate whether the addition of rPRT to genotypic resistance testing (GRT) compared to GRT alone is beneficial for obtaining a virological response in heavily pre-treated HIV-infected patients.

**Methods:**

Patients with resistance tests between 2002 and 2006 were followed within the Swiss HIV Cohort Study (SHCS). We assessed patients' virological success after their antiretroviral therapy was switched following resistance testing. Multilevel logistic regression models with SHCS centre as a random effect were used to investigate the association between the type of resistance test and virological response (HIV-1 RNA <50 copies/mL or ≥1.5log reduction).

**Results:**

Of 1158 individuals with resistance tests 221 with GRT+rPRT and 937 with GRT were eligible for analysis. Overall virological response rates were 85.1% for GRT+rPRT and 81.4% for GRT. In the subgroup of patients with >2 previous failures, the odds ratio (OR) for virological response of GRT+rPRT compared to GRT was 1.45 (95% CI 1.00-2.09). Multivariate analyses indicate a significant improvement with GRT+rPRT compared to GRT alone (OR 1.68, 95% CI 1.31-2.15).

**Conclusions:**

In heavily pre-treated patients rPRT-based resistance information adds benefit, contributing to a higher rate of treatment success.

## Background

Combination antiretroviral therapy (cART) has dramatically reduced HIV related morbidity and mortality. Potent new drugs for patients with multiple drug resistance have been introduced [1-5]. Nevertheless, virological failure in treatment-experienced patients is still a major concern and therefore HIV drug resistance testing has a key role for the optimal choice of active drugs in patients with multiple drug failure. Accordingly, current guidelines recommend resistance testing for patients with multiple drug failure, but also for newly infected individuals and for pregnant women as transmission of resistant HIV mutants to therapy naïve individuals are a rising concern [6-9].

Two technical principles are in use today for resistance testing: Genotypic resistance tests (GRT) and phenotypic resistance tests (PRT). GRT is based on population gene sequencing of defined DNA segments, typically to detect mutations, which represent at least 20% of the virus population and confer HIV-1 drug resistance [10,11]. As a special form of genotyping, virtual PRT (vPRT) correlates genotypic data for plasma HIV-1 RNA of a candidate gene with a large database of paired biological and clinical phenotypes [12-15]. Numerous genotypic interpretation systems have become available during the past decade, which provide excellent prediction of drug response. On the other hand, comparing different algorithms, some very significant differences and opposite predictions continue to be observed for the interpretation of the impact of mutational patterns (T. Klimkait, manuscript in preparation).

PRT assesses viral expression. A special form of it, the replicative phenotypic resistance test (rPRT) utilizes several replication cycles of a recombinant infectious virus to follow viral propagation in the presence of antiretroviral drugs [16,17]. By permitting several cycles of viral replication in vitro rPRT can detect viral minorities below one percent [18]. However, rPRT is more costly, and takes longer than GRT.

Several studies have demonstrated the clinical benefit and cost-effectiveness of GRT [19-25] compared to standard of care. This study was designed to analyse whether the dissecting, sensitive format of PRT may provide a diagnostic benefit over GRT. Analyses comparing virtual PRT to GRT have thus far not been able to document a clear clinical advantage for PRT with a higher proportion of patients achieving a suppressed viral load [14,15,26-30]. Our first retrospective single centre analysis of GRT combined with a highly sensitive rPRT already suggested, although statistically underpowered, that patients being switched to new cART based on drug choice from a combination of both tests tended to have better virological response than those with only GRT-based resistance information [31].

In the present study we included all available data of prospectively conducted resistance tests for patients enrolled in the much larger multicentre Swiss HIV Cohort Study (SHCS) and compared the virological outcome in patients initiating a new antiretroviral drug regimen based on results of either GRT alone or rPRT combined with GRT. The highly sensitive format of rPRT used in the SHCS allows the detection of less than 1% of resistant virus in a clinical sample with a mixed virus population [18]. We therefore explored whether the complementing information of rPRT improves patient outcome when used routinely in the clinical setting.

## Methods

### Study population

The SHCS is a prospective cohort study with continuing enrollment of HIV-infected individuals aged 16 years or older [32]. The Swiss HIV cohort study has been approved by ethical committees of all participating institutions. Written informed consent has been obtained from all participating patients. Clinical visits take place every six months at seven outpatient clinics of participating HIV-centres, associated hospitals, or specialized private doctors' offices. Any request for a resistance test as well as information on indication and outcome of current and previous therapies are recorded in the central database of the SHCS. Individuals who had a prospective resistance test performed between 2002 and 2006 for which the physician had access to results prior to making clinical decisions were eligible for the study if the following criteria were fulfilled; (i) cART was changed within one year after a resistance test was performed, (ii) the patient was off treatment for <6 months following the resistance test before starting a new regimen and (iii) at least one HIV-1 viral load measurement was available following the switch of antiretroviral therapy. Patients on any protocol for structured treatment interruption studies were excluded. In situations where multiple resistance testing was done only the first eligible test for an individual was utilized. Patients were followed from the time of the switch to a new cART regimen following resistance testing to the earliest of any of the following events: switch to a new cART regimen due to virological failure, going off treatment, death, loss to follow-up, or the closing date of the study, July 31, 2008.

The reason for resistance testing has to be provided by the clinician ordering a given resistance test. The specified categories for resistance testing are: drug naive prior to initiation of first therapy, primary infection, suspicion for resistant virus transmission, pregnancy, and drug failure. The indication "primary infection" is specified by characteristics of very early infection stages with skin rash, very high virus load and incomplete immunoreactivity; "resistant virus transmission" is indicated when high promiscuity or the involvement of highly therapy-experienced individuals is suspected. When the reason for testing was missing, we utilized information from the SHCS to classify patients. Individuals were considered to have had testing for drug failure if they had either RNA >1000 copies/mL, 1-2 previous ART regimens and RNA between 500-1000 copies/mL, or were on a salvage therapy (>2 previous ART regimens).

GRT is performed in Switzerland in four dedicated laboratories of the SHCS that use different techniques [33,34]. One centre uses an in-house test, one uses the VircoTYPE HIV-1 Assay (Virco Laboratory, Mechelen, Belgium), and two use the ViroSeq System (Abbott AG, Baar, Switzerland)

The rPRT system used in Switzerland is based on a position-precise ligation of patient-derived PR/RT sequences into a replication competent background of a standardized reference HIV-1. As the entire amplified virus population is retained during DNA plasmid propagation this process represents to the best extent possible the virus population present in a patient's blood at the time of blood draw. The subsequent introduction of the DNA plasmids into susceptible human reporter cells initiates a rapid HIV infection. The diagnostic system, termed deCIPhR/PhenoTecT, allows the reconstituted HIV to undergo in a time window of four days 3-4 rounds of replication in the presence of each drug separately. A first replication round in this system thereby eliminates any susceptible wild type viruses, while relevant drug resistant variants are amplified during several cycles. A stably integrated LTR-driven reporter is activated by HIV Tat, and its expression has been shown to directly correlate with cellular HIV infection [35]. The deCIPhR system has been demonstrated to detect resistant variants present at less than 1% in the viral population and is able to dissect mixed virus populations. The short assay duration (6 days) obviates de novo evolution of resistance in vitro. Details and a comparison with non-replicative systems have been described earlier [16-18].

### Outcome definition and main predictor

The primary endpoint of the study was virologic response defined as either HIV-1 RNA viral load <50 copies/mL or a reduction in viral load of ≥1.5 log copies/mL. Once an individual started the new cART regimen, any further regimen switches prior to achieving virological response were defined as a failure unless no HIV-1 RNA was measured.

Our main predictor was the type of resistance testing an individual received: GRT alone or GRT plus rPRT. The following covariates were considered for inclusion in the analysis to adjust for potential confounding: age (<40, ≥40 years), gender, current intravenous drug use (IDU) or participation in a drug maintenance program, HIV-1 RNA (log_10 _transformed), nadir CD4 cell count (square root transformation, per 100 cells per μL), number of previous cART regimens, cART regimen class, calendar year and adherence to antiretroviral drugs (maximum number of self-reported missed cART-doses in the 4 weeks prior to a cohort visit) [36].

### Statistical methods

Baseline characteristics of the eligible population were summarized overall and by resistance test. We explored whether rPRT in addition to GRT was associated with higher rates of virological response. To study the effects of the type of resistance test on the success of therapy, multilevel logistic regression analysis was performed. SHCS centre was included in the model as a random effect to account for the potential higher correlation in response among individuals seen at the same centre.

Based on our hypothesis that the benefit of rPRT would be greatest in those with previous drug failure, we pre-defined two subgroups for additional analysis: patients having a resistance test after any treatment failure and patients having a resistance test after >2 previous treatment failures.

The association between explanatory variables and treatment success were assessed by odds ratios (OR) and 95% confidence intervals (CI); OR above 1 indicate that a covariate is positively associated with the outcome. All analyses were done with SAS 9.1 (SAS Institute, Cary, North Carolina, USA). The manuscript was written to comply with STROBE (Strengthening the reporting of observational studies in epidemiology) guidelines [37].

## Results

### Baseline characteristics

For the period 2002-2006 we identified 2268 individuals with a total of 2889 resistance test samples. Of these, 1459 tests from 1204 individuals were excluded. The reasons for ineligibility were no change of cART following resistance testing (49.0%), a change of cART later than one year following resistance testing (36.1%), patients being off cART for more than 6 months following resistance testing (8.6%), and 6.7% with no available HIV-1 RNA viral load following resistance testing (Table 1). The high percentage of the "no change" category reflects a combination of those cases where primary infections were analyzed, or patients after deliberate therapy interruption, or those with imperfect therapy compliance. Consequently no treatment adjustment occurred.

**Table 1:** Exclusion criteria for comparison of GRT versus GRT + rPRT

	All N (%)	GRT N (%)	GRT + rPRT N (%)
**Ineligible tests - n (% of total tests)**	**1459**	**1120**	**339**

No change of ART after RT	708 (49.0)	532 (47.5)	176 (51.9)

Change of ART only >1 year after last RT	526 (36.1)	411 (36.7)	116 (34.2)

Off treatment for >6 months after RT	126 (8.6)	105(9.4)	21 (6.2)

No RNA during study period*	98 (6.7)	74 (6.6)	25 (7.4)

Other#	1 (0.01)	0.0	1 (0.3)

* The study period is the time from the 1^st ^change of ART after RT until the earliest of either changing ART due to failure (RNA >400), going off treatment, or December 31, 2008.

# Participation in a structured treatment interruption trial.

RT = resistance test, GRT = genotype RT, rPRT = replicative phenotype RT, cART = combined antiretroviral therapy

The final study population consisted of 1158 individuals, with their corresponding resistance tests. Of these 1158 individuals, 937 received GRT and 221 GRT plus rPRT. The indication for the resistance test was drug failure (66.5%), testing for transmission of resistant viruses in naïve patients (28.5%), pregnancy (3.5%), and unknown (1.5%). There was no relevant difference in the distribution of the indication for resistance testing according to the type of resistance test (Table 1).

Table 2 shows the baseline characteristics of the study population overall and by type of resistance test. The median age was 41 years (median, inter-quartile range (IQR) 36-47 years), 69.4% were men, 29.2% had a previous AIDS diagnosis and 12% of all subjects were current IDU or in a drug substitution program at that time. At baseline (time of resistance testing) HIV-1 RNA was 4.2 log copies/mL (median, IQR: 3.2-4.9 log copies/mL) and the median CD4 cell count was 261 cells/μL (IQR: 168-387). Of note, 30.2% of the population was drug naïve and 55.5% currently on therapy with a median of two previous cART (IQR: 0-6) regimens. Because of differences in the local availability of RT across the SHCS centres, the use of rPRT differed substantially with 2 centres contributing over 80% of rPRT and 2 centres not performing rPRT at all.

**Table 2:** Patient characteristics of HIV-infected individuals according to type of resistance test (RT)

	All	GRT	GRT + rPRT
Total tests - n	1158	937	221

Male - %	69.4	68.8	72.0

Age - median [IQR]	41 [36-47]	41 [36-47]	40 [36-47]

Caucasian - %	79.5	79.9	77.8

HIV transmission group - %			
Homosexual	39.5	40.7	34.4
Heterosexual	38.0	37.3	41.2
IDU	18.7	18.6	19.5
Other	3.8	3.5	5.0

Current IDU or in drug maintenance program - %	12.0	11.9	12.7

Baseline HIV-1 RNA (copies/mL)†- %			
Log RNA - Median [IQR]	4.2 [3.2-4.9]	4.2 [3.2-4.9]	4.2 [3.3-4.9]
= 50	2.6	3.0	0.9
51 - 500	10.6	10.6	10.7
501 - 1000	5.0	5.0	5.1
>1000	81.8	81.4	83.3

Baseline CD4 cell count (109)† - %			
Median [IQR]	261 [168-387]	260 [166-387]	266 [180-390]
<200	33.1	33.4	32.1
200 - 349	36.5	36.8	34.9
350 - 499	17.9	17.4	20.1
= 500	12.6	12.5	12.9

Hepatitis C¶ - %	3.2	3.8	0.5

AIDS - %	29.2	29.4	28.5

Number of previous ART regimens			
Median [IQR]	2 [0-6]	2 [0-6]	2 [0-5]

Treatment status at time of RT - %			
Naïve	30.2	29.5	33.5
Off treatment	14.3	15.5	9.1
Current	55.5	55.1	57.5

ART after RT - %			
NNRTI	26.3	24.8	32.6
PI non-boosted	5.9	5.9	5.9
PI boosted	58.8	60.7	50.7
Triple Nucleoside/Other	9.1	8.6	10.9

Maximum missed doses of ART# -%			
0	49.9	51.2	45.9
1	15.1	13.5	20.2
2	12.9	13.5	11.0
>2	22.2	21.9	22.9

Missed 2 consecutive doses of ART# - %	19.2	19.1	19.4
SHCS centre at time of RT - %			
Basel	12.2	3.0	51.1
Bern	14.8	10.8	31.7
Geneva	19.3	23.8	0
Lausanne	10.0	12.4	0
Lugano	3.1	3.4	1.8
St. Gallen	4.2	2.2	12.2
Zurich	36.5	44.4	3.2

¶ Active/chronic hepatitis C

† Baseline is the time of RT. Labs closest to before or after the RT.

# In the year prior to RT.

RT = resistance test, GRT = genotypic RT, rPRT = replicative phenotypic RT, ART = antiretroviral therapy, IQR = interquartile range, IDU = intravenous drug use,

### Primary endpoint: virological response after resistance test

All patients had a minimum of one year of follow-up in this study. This was considered a sufficiently long period for achieving virological success on a new regimen even in situations where patients had been heavily pre-treated. Following resistance testing 81.4% (n = 763 of 937) in the GRT group and 85.1% (n = 188 of 221) in the combined GRT plus rPRT group achieved the primary endpoint of virological response (either VL <50 copies/mL or 1.5 log reduction). The type of success achieved did not vary by type of resistance test with 51.4% of those with GRT and 49.5% of those with GRT plus rPRT achieving a VL <50 copies/mL. Success rates for GRT and GRT plus rPRT in the subset with resistance testing due to failure were 74.4% and 79.7%, in salvage patients 69.0% and 77.5%, respectively. The OR in univariable multilevel logistic regression analysis for virological response of GRT plus rPRT compared to GRT was 0.85 (95% CI 0.59-1.24) and for the pre-specified subgroups of patients with any and >2 previous drug failures were 1.16 (95% CI 0.73-1.82) and 1.45 (95% CI 1.00-2.09), respectively (Table 3).

**Table 3:** Multi-level univariable logistic regression models for virological response in patients with GRT+rPRT compared to GRT *

	n	OR (95% CI)	p-value
All patients	1158	0.85 (0.59 - 1.24)	0.41

Patients with any failure	770	1.16 (0.73 - 1.82)	0.53

Patients with >2 previous failures	533	1.45 (1.00 - 2.09)	0.05

* Models are hierarchical with follow-up centre included as a random effect. Virological response is defined as a reduction by ≥1.5 log HIV-1 RNA viral load or less than 50 copies/mL

For the pre-specified subgroup of patients with >2 previous drug failures this association was highly significant in multivariate analysis when adjusting for age, gender, IDU, baseline HIV-1 RNA, CD4 nadir, number of previous regimens, class of cART, and missed doses of cART (OR 1.68, 95% CI 1.31-2.15) (Table 4). The CD4 nadir, class of cART regimen and self-reported missed cART doses remained significant predictors of virological response in this subgroup of patients. As also shown in table 4 a lower number of patients in the GRT group remained on NNRTI-containing regimens and, in contrast, a higher percentage received the newer, seemingly more potent PI-based therapies.

**Table 4:** Multi-level logistic regression models for virological response in patients with >2 previous failure (n = 533) with GRT+rPRT compared to GRT *

Model	Univariate OR (95% CI)	Multivariate OR (95% CI)	Adjusted p-value
Type of resistance test (GRT+rPRT vs. PRT)	1.45 (1.00 - 2.09)	1.68 (1.31 - 2.15)	<0.001

Age (≥40 vs. <40)	1.10 (0.74 - 1.64)	1.22 (0.90 - 1.65)	0.20

Male	0.77 (0.45 - 1.32)	0.80 (0.40 - 1.61)	0.53

Current IDU or in drug maintenance programme	1.27 (0.58 - 2.78)	1.94 (0.97 - 3.90)	0.06

Baseline HIV RNA (log10 copies/mL)	0.83 (0.66 - 1.05)	0.87 (0.64 - 1.18)	0.37

CD4 nadir (square root per 100 cells/μL)	1.66 (1.18 - 2.32)	1.67 (1.16 - 2.41)	0.006

Number of previous ART regimens	0.94 (0.88 - 1.00)	0.95 (0.90 - 1.00)	0.07

ART regimen after RT test			
NNRTI	Reference	Reference	
PI non-boosted	0.12 (0.02 - 0.78)	0.13 (0.03 - 0.64)	0.01
PI boosted	0.31 (0.16 - 0.59)	0.43 (0.23 - 0.79)	0.007
Triple nucleoside/Other	0.08 (0.02 - 0.28)	0.08 (0.02 - 0.27)	<0.001

Missed doses#			
0	Reference	Reference	
1	0.52 (0.27 - 0.98)	0.42 (0.22 - 0.82)	0.01
2	0.41 (0.16 - 1.05)	0.41 (0.14 - 1.22)	0.11
>2	0.41 (0.29 - 0.58)	0.37 (0.24 - 0.57)	<0.001

* Models are hierarchical with follow-up centre included as a random effect. Virological response is defined as HIV-1 RNA viral load <50 copies/mL or reduction by ≥ 1.5 log

# Maximum number of missed doses during the study period

GRT = genotypic RT, rPRT = replicative phenotypic RT, OR = odds ratio, CI = confidence interval, IDU = injecting drug use

The new potent drugs such as darunavir and etravirine were not yet marketed in Switzerland. Nevertheless, calendar year was considered as a possible confounder in the model. Yet it was not found to be a relevant variable. When adding it to the multivariable model in Table 4, the odds ratio for type of resistance test remained unchanged (OR: 1.68, 95% CI: 1.37-2.04).

## Discussion

In this multicentre cohort study of prospectively assessed HIV-1 drug resistance in patients the addition of rPRT to GRT as compared to GRT alone showed a trend towards improved success rates for treatment with increasing levels of antiretroviral pre-treatment. In the subgroup of heavily pre-treated patients with multiple drug failures the addition of rPRT significantly improved virological outcome with a 70% increased odds for achieving treatment success after adjusting for confounders and SHCS centre. The clinical benefit of resistance testing must be critically evaluated in its clinical context. Between 1999 and 2007 resistance declined overall in the SHCS [38]. This decrease was mainly driven by two mechanisms, the loss to follow-up or death of high-risk patients exposed to mono- or dual-nucleoside reverse transcriptase inhibitor therapy and the continued enrolment of low risk patients who were taking cART that contained boosted protease inhibitors or NNRTI as first-line therapy.

From a virologist's point of view the add-on benefit of rPRT is of particular relevance in patients with multiple drug failure and archived mutations. In patients with multiple virological drug failure and multiple therapy changes the genomic complexity of deposited HIV sequences increases. Growing resistance coincides with a rise of viral quasispecies [18,39,40]. Although GRT provides relevant information to clinicians for optimal drug choices it has important limitations for mixed virus populations and for the detection of emerging or residual virus variants. The interpretation of a GRT results becomes particularly challenging for therapy-experienced patients where specific mutations have to be assigned to distinct HIV genomes. Today several unique rule based algorithms are very well established e.g. Stanford (HIV drug resistance database, Stanford university; USA), ANRS (National Agency for AIDS Research, France), Rega (Institute for Medical Research and University Hospitals, Belgium), and G2P (geno2pheno system, Max-Planck-Institute, Germany). However, the agreement among these algorithms tends to decrease in parallel to the growing complexity of viral mutation patterns [41]. Interpretation and choice of the optimal regimen becomes particularly difficult for heavily pretreated patients, where the clinical treatment options become scarce or in situations where drug pressure after longer treatment interruptions is absent.

One intrinsic potential limitation of this study lies in the fact that the choice of requesting GRT or GRT+PRT was largely centre-dependent, thereby introducing a possible centre bias and depending on any centre's preference for certain regimens. However by using a multilevel or hierarchical model, the effect of resistance testing was estimated after adjusting for the measured or unmeasured effect of centre.

Our study has several strengths. We used stringent and very conservative criteria to define the target population of this observational cohort study. The cohort represents an unselected population of HIV-infected individuals, which is larger than the populations included in previously published observational studies and clinical trials. In addition this study includes a relatively large number of females and IDU making it more representative. We were able to include important variables in our analysis known to be related to virological outcome. For example, our data indicate that the study population included a relatively large group of patients with adherence problems in comparison to the general patient population in the SHCS. Roughly one third of patients had indicated that they had missed more than 2 doses in the previous four weeks and one fifth of patients stated to have missed more than 2 consecutive doses. Thus, our findings should be interpreted in the context of a patient population that poses real challenges for optimal clinical management and most likely makes it more difficult to demonstrate an add-on benefit of rPRT to GRT than one would have seen in a clinical trial with a more selected patient population.

High molecular diversity of HIV is a common problem in long-term treated, highly therapy experienced patients. In such patients with complex resistances rPRT is able to assign resistances to several co-existing viruses rather than placing the gene mutations onto one single virtual virus genome as done by GRT, and thus gives more conservative estimates of antiretroviral drug resistance. In contrast, GRT in such patients leads to over-simplification by indicating cross resistance patterns per viral genome that tend in reality to be more complex. As a consequence, GRT may in these patients over-interpret the viral resistance and erroneously indicate to clinicians and their patients a lower number of remaining treatment options. The higher percentage in the GRT group of PI containing regimes, paralleled by a reduction in NNRTI-containing ART combinations suggests two likely reasons: on the one hand a centre effect for the favoured therapy-scheme, on the other, due to their low genetic barrier, the prompt stop of NNRTIs after virological failure. This study did, however, not assess whether or not this decision was always based on the formal demonstration of predominant NNRTI-related resistance mutations.

Previous studies on GRT and PRT have investigated virological outcome with mixed findings, either resulting in non-significant gains [19-21] or in only a small benefit [25] and cost savings [22-24] from GRT-guided therapy adjustment. Moreover, several clinical trials have investigated different types of PRT, but until now a possible advantage of providing PRT remains unclear. In a randomized trial of heavily pre-treated patients PRT did not result in an intention to treat analysis in a greater proportion of virological suppression when compared to standard of care. In the as treated analysis a statistically significant 16% difference of patients with less than 400 c/mL was found [26]. In another randomized trial by Meynard et al. a less sensitive single-cycle PRT was used. The combination of PRT with GRT compared to GRT did not result in a higher rate of HIV-1 suppression [28]. GRT plus vPRT was compared to GRT in a large Australian trial but the investigators found at 48 weeks no difference in virological outcome [27]. In one trial patients with drug failure were randomized either to access to routine GRT, vPRT, or "no testing". No difference in the time to virological failure was found between groups. However, in the subgroup of patients with more than four previous failures patients with vPRT did have significantly prolonged time to treatment failure [29]. In another randomized controlled trial by Dunn et al. there was no difference between GRT alone and GRT plus PRT [30]. Both trial groups worked with a less sensitive method of PRT compared to the one used in this study.

## Conclusion

Evidence from clinical trials investigating whether GRT, PRT or the combination of both improve virological outcome is limited. Subgroup analyses from trials suggest that PRT may improve clinical outcome in patients with multiple previous failure. Our findings are in line with those trials. Our study shows that rPRT, when added to GRT, may indeed lead to improved virological outcome, particularly in the population of heavily pre-treated patients. This is corroborated by the finding that a therapy status "no treatment at time of testing" is significantly less frequent for the GRT + rPRT group. This indicates that GRT + rPRT was more often chosen in complex therapy situations. As scientific basis: replicative PRT functionally dissects resistant virus populations and may reveal remaining viable regimens, particularly in patients with limited options and thereby increase the chance for virological success. In contrast GRT tends to place for analysis all mutations on one viral "consensus" genome.

Our study suggests that a stepwise testing strategy adding replicative PRT for patients with multiple drug failure provides benefit for better clinical decision-making. Further studies are needed to confirm whether this strategy translates into improved virological outcome in patients with limited treatment options.

## Competing interests

The authors declare no competing interests. During the study period Th. Klimkait was part-time employee at InPheno AG, Basel.

## Authors' contributions

JF and TK conceived the study, participated in its design and coordination and wrote the manuscript. TG and HB carried out the statistical analysis and were also involved in the main writing process of the manuscript. SL and FH were responsible for the performance of the genetic and phenotypic laboratory resistance test analysis. HH, VW, JB, SY, PB, MC, CF, BH, PV, GL, EB, HG and MB were involved in clinical and laboratory data collection in their respective clinical centres and in interpretation of the data and participated in the review of the final manuscript.
